# Inflammation and In-Stent Restenosis: The Role of Serum Markers and Stent Characteristics in Carotid Artery Stenting

**DOI:** 10.1371/journal.pone.0022683

**Published:** 2011-07-28

**Authors:** Katrin Wasser, Sonja Schnaudigel, Janin Wohlfahrt, Marios-Nikos Psychogios, Michael Knauth, Klaus Gröschel

**Affiliations:** 1 Department of Neurology, University of Göttingen, Göttingen, Germany; 2 Department of Neuroradiology, University of Göttingen, Göttingen, Germany; 3 Department of Diagnostic Radiology, University of Göttingen, Göttingen, Germany; Julius-Maximilians-Universität Würzburg, Germany

## Abstract

**Background:**

Carotid angioplasty and stenting (CAS) may currently be recommended especially in younger patients with a high-grade carotid artery stenosis. However, evidence is accumulating that in-stent restenosis (ISR) could be an important factor endangering the long-term efficacy of CAS. The aim of this study was to investigate the influence of inflammatory serum markers and procedure-related factors on ISR as diagnosed with duplex sonography.

**Methods:**

We analyzed 210 CAS procedures in 194 patients which were done at a single university hospital between May 2003 and June 2010. Periprocedural C-reactive protein (CRP) and leukocyte count as well as stent design and geometry, and other periprocedural factors were analyzed with respect to the occurrence of an ISR as diagnosed with serial carotid duplex ultrasound investigations during clinical long-term follow-up.

**Results:**

Over a median of 33.4 months follow-up (IQR: 14.9–53.7) of 210 procedures (mean age of 67.9±9.7 years, 71.9% male, 71.0% symptomatic) an ISR of ≥70% was detected in 5.7% after a median of 8.6 months (IQR: 3.4–17.3). After multiple regression analysis, leukocyte count after CAS-intervention (odds ratio (OR): 1.31, 95% confidence interval (CI): 1.02–1.69; p = 0.036), as well as stent length and width were associated with the development of an ISR during follow-up (OR: 1.25, 95% CI: 1.05–1.65, p = 0.022 and OR: 0.28, 95% CI: 0.09–0.84, p = 0.010).

**Conclusions:**

The majority of ISR during long-term follow-up after CAS occur within the first year. ISR is associated with periinterventional inflammation markers and influenced by certain stent characteristics such as stent length and width. Our findings support the assumption that stent geometry leading to vessel injury as well as periprocedural inflammation during CAS plays a pivotal role in the development of carotid artery ISR.

## Introduction

Cerebral ischemia is one of the most important causes of mortality in industrialized countries and is associated with considerable medical and socio-economic problems [1]. Carotid artery stenosis is known as a leading risk factor for the development of ischemic stroke and is therefore a major target of primary and secondary stroke prevention. Randomized controlled trials and subsequent meta-analyses have demonstrated that carotid endarterectomy (CEA) in combination with best medical treatment of cerebrovascular risk factors is currently the standard treatment for patients with a symptomatic carotid artery stenosis and some selected patients with an asymptomatic carotid artery stenosis [2]. During the last decade, carotid angioplasty and stenting (CAS) has emerged as an alternative treatment modality and may be used as a complementary treatment to CEA [3]–[5]. As has been indicated within the recently published pooled analysis of the EVA-3S, SPACE, and ICSS studies as well as in the CREST study, CAS may be a safe treatment option especially in patients aged <70 years [6], [7]. However, the efficacy of CAS in younger patients is highly dependent on its long-term outcome. Unfortunately, prospective long-term data are sparse and the current advantages and complications of CAS are still controversially debated. Particularly, the long-term patency of carotid artery stents may be endangered by a pathological neointimal proliferation and subsequent in-stent restenosis (ISR). ISR is frequently asymptomatic at diagnosis, but may nevertheless adversely affect the long-term safety and efficacy of CAS because it could necessitate a second intervention which might again be associated with a periprocedural complication.

We know from coronary artery stenting that inflammation plays a pivotal role in the pathogenesis of ISR, causing neointimal proliferation through the stent meshes [8]–[10]. In this scenario, technical factors such as stent dimensions or pre- and postdilation during CAS may result in a vascular burden due to vessel injury. This could cause inflammation and thus contribute to the development of an ISR after CAS due to neointimal proliferation. In vivo, an inflammatory process can easily be monitored with inflammatory serum biomarker for instance C-reactive protein (CRP), which has shown its predictive value in the clinical and imaging outcome of patients undergoing coronary and carotid artery stenting [11]–[13].

The aim of the current study was to investigate the influence of periprocedural serum inflammatory biomarkers and procedural technical characteristics on the incidence of ISR in patients undergoing CAS during long-term follow-up.

## Methods

### Ethics statements

The current study is in accordance with the Declaration of Helsinki and ICH/GCP guidelines. The analysis was approved by the Ethics Committee of the University of Göttingen, Germany.

### Study design

197 patients (215 arteries) with a symptomatic carotid artery stenosis ≥70% or an asymptomatic carotid artery stenosis ≥90% (degree of stenosis was measured according to the European guidelines (ECST) [14]) who underwent carotid artery stenting between May 2003 and June 2010 and were prospectively examined at our institution were consecutively included in our study. Exclusion criteria for this current analysis were unstable neurological conditions, progressive stroke, stenosis caused by dissection or markedly elevated CRP levels or leukocyte counts preinterventionally due to manifest infections such as pneumonia or urinary tract infections.

Patients who had experienced a transient or permanent ipsilateral ocular or cerebral ischemic event within the past six months due to carotid artery stenosis were considered symptomatic. The degree of stenosis was determined by carotid duplex ultrasound imaging according to the ECST guidelines and angiographically confirmed during the stenting procedure. All patients received detailed information about the three different treatment strategies (CEA, CAS, and best medical treatment) and their specific advantages and potential complications. With respect to the CAS procedure, all patients were informed about the investigational nature of CAS and gave their written informed consent.

### Clinical data collection

An experienced stroke neurologist (K.W., S.S. or K.G.) performed a complete neurological examination before the procedure, immediately after CAS and at every visit during long-term follow-up, and documented all clinical data. The following cerebrovascular risk factors were recorded using history or direct measurements: hypertension (blood pressure ≥140/90 mmHg measured on repeated occasions or presence of antihypertensive drugs), hyperlipidemia (fasting serum cholesterol levels ≥200 mg/dl or statin therapy), smoking (current or within the previous year), diabetes mellitus (HbA1c ≥6.5%, fasting blood glucose ≥120 mg/dl, or presence of antidiabetic drugs), coronary artery disease (history of angina, myocardial infarction, percutaneous transluminal angioplasty or surgery), peripheral occlusive arterial disease (history of typical clinical presentation, percutaneous transluminal angioplasty or surgery) and the presence of contralateral carotid artery stenosis or occlusion (as assessed with ultrasound).

### Imaging methods

**Figure 1:**
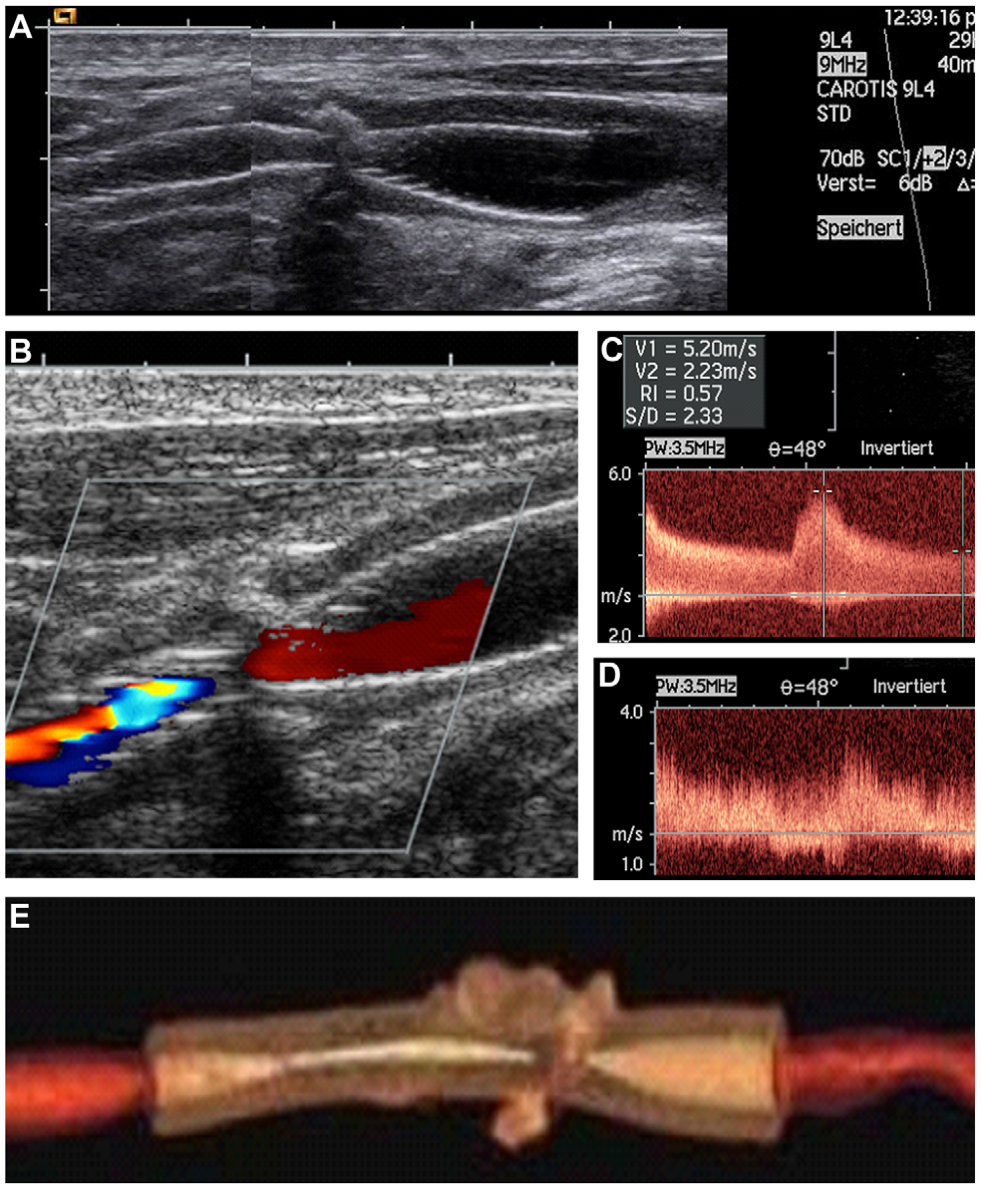
In-stent restenosis after carotid artery stenting as diagnosed during routine duplex sonography follow-up. Duplex sonography (B-mode) of a carotid artery after stenting showing a narrowing in the middle part of the stent due to a calcified plaque (A). During the routine follow-up investigation after six months there was a typical aliasing phenomenon indicating focal flow acceleration (B). Peak systolic velocity reached up to 520 cm/s (C) with a markedly disturbed poststenotic frequency pattern (D). The reconstructed contrast enhanced computer tomography confirmed the high-grade in-stent restenosis (E).

Carotid duplex ultrasound imaging using a combination of direct and indirect criteria as well as the presence and extent of intrastenotic and poststenotic flow yielded the degree of stenosis before CAS and the diagnosis of an ISR. In detail, as direct criteria for the local degree of stenosis, the peak systolic flow velocities (PSV) within and distal of the carotid artery stenosis, the peak enddiastolic flow velocity in the stenosis, the internal carotid artery-to-common carotid artery PSV ratio, and the prestenotic and poststenotic frequency patterns were documented. Whenever possible, the residual vessel lumen in the B image and the colour-coded residual vessel area were determined. As indirect criteria, a reversal flow of the supratrochlear artery and the anterior cerebral artery as an indicator of an inadequate intracerebral crossflow and the pulsatility of the ipsilateral common carotid artery were taken into account. As there are no common criteria available for defining an ISR and current literature suppose different criteria [15]–[17], we used locally adopted criteria with a PSV ≥300 cm/s as a key feature representing an ISR of ≥70% (Figure 1).

All examinations were performed in a standardized form in the same vascular laboratory with the same ultrasound equipment (Acuson Sequoia™ 512, Siemens, San José, CA) under the supervision of an experienced, board certified vascular neurologist (K.G.).

Contrast-enhanced reference imaging (computer tomography angiography and/or conventional angiography) was done at the discretion of the treating physician, in most of the cases if the collateral flow pattern changed, in case of the occurrence of clinical stroke-symptoms attributable to the treated vessel or if a re-intervention was anticipated.

### Laboratory data collection

Peripheral blood samples for routine measurement of CRP and leukocyte count were taken 24 hours before and after CAS. Furthermore, a serum lipid profile containing high density lipoprotein (HDL), low density lipoprotein (LDL) and total cholesterol as well as triglycerides were tested during hospitalization after overnight fasting.

All parameters were determined by standard laboratory methods at the Department of Clinical Chemistry of the University Medical Center of the Georg-August-University Göttingen, Germany. In detail, C-reactive protein was assayed by an automated immunoprecipitation technique with photometric analysis. The reference range of our local laboratory is ≤8.0 mg/l, the lower detection limit 0.2 mg/dl. The leukocyte count was also measured sample dependent by automated techniques with one of the following haematology analyzers: Beckman Coulter AcT 5 diff AL (Beckmann Coulter, Brea, CA, USA), ADVIA® 120 (Bayer Diagnostics, München, Germany) or Abbot Cell-Dyn Sapphire (Diamond Diagnostics, Holliston, MA, USA) with a local reference range of 4000–11000/µl. A following manual count was carried out if pathological results occurred.

Serum triglycerides, total cholesterol and HDL cholesterol were measured in fasted blood samples using an autoanalyzer. LDL cholesterol was calculated using the Friedwald's formula [18].

### CAS procedure

In the majority of cases, the intervention was done under anaesthesiology stand-by via a femoral approach by an experienced interventional neuroradiologist. Stent-type and the use of filter-based protection devices were chosen at the discretion of the interventionalist. All patients received orally administered aspirin (100 mg/d) and clopidogrel (75 mg/d) at least 3 days before the procedure. Aspirin was administered indefinitely and clopidogrel was withdrawn 6 weeks after CAS. All patients were routinely monitored in our intensive care or stroke unit overnight and were discharged to normal ward or home thereafter. A complete neurological status of the patient and a duplex sonography was performed before discharge.

### Follow-up protocol

A neurologist experienced in neurovascular diseases examined each patient and recorded clinical complications at the hospital's outpatient clinic at 3, 6, and 12 months after the CAS-procedure and every 6 months thereafter. During these postinterventional visits serial duplex sonography was routinely performed according to a standardized protocol.

### Statistical analysis

Continuous values were expressed as mean ± SD and nominal variables as count and percentages. Median values with the corresponding interquartile range (IQR) were computed for non-normally distributed variables. A two-sided T-test was used for comparison of normally distributed variables and the non-parametric Kruskal Wallis test for not normally distributed values. For comparisons of categorical data we used two-tailed Chi-square statistics with Yates' correction or the Fisher's exact test when the predicted contingency table cell values were less than five. A two-sided p value of less than 0.05 was considered to indicate a statistically significant difference. Multiple binominal regression analysis was conducted for those variables with a p<0.1 on univariate level to estimate a potential effect on the development of an ISR (p to enter = 0.05, p to leave = 0.1) using additive and multiple interaction terms. All statistical analyses were performed using SPSS (Version 17, SPSS Inc., Chicago, Ill).

## Results

### Patient characteristics and follow-up

We retrospectively screened 215 patients (237 arteries) consecutively undergoing elective carotid artery stenting between May 2003 and June 2010 from our prospectively achieved database. The data of 21 patients (22 arteries) had to be excluded due to missing follow-up data, additionally 5 patients (5 arteries) with clinical signs of pulmonary or urinary tract infections and/or markedly elevated CRP levels or leukocyte counts were excluded. Complete clinical follow-up data were available for the remaining 210 CAS procedures (mean age of 67.9±9.7 years, 71.9% male), with a median duration of follow-up of 33.4 months (IQR: 14.9–53.7 months). A PSV ≥300 cm/s corresponding to an ISR ≥70% was detected in 12/210 (5.7%) arteries after a median of 8.6 months (IQR: 3.4–17.3). In 9 of the 12 ISR a new crossfilling via the anterior communicating artery or supraorbital artery could be identified as additional supporting sonographic criteria for a high grade restenosis. A contrast-enhanced reference imaging confirmed the ISR (see Figure 1) in nine cases (seven by conventional angiography, two by CT angiography), failed in one case (CT angiography) and was not done in two cases. The detailed clinical baseline patient characteristics are given in table 1.

**Table 1:** Baseline patient characteristics (n = 210).

Variable	Data
Mean age (years ± SD)	67.9 (±9.7)
Male sex	151 (71.9%)
Right sided carotid stenosis	89 (44.3%)
Symptomatic carotid stenosis	149 (71.0%)
Arterial hypertension	191 (91.0%)
Hyperlipidemia	140 (66.7%)
Tobacco use	63 (30.0%)
Diabetes mellitus	62 (29.5%)
Coronary artery disease	61 (29.0%)
Peripheral occlusive arterial disease	41 (19.5%)
Contralateral carotid occlusion	28 (13.3%)
Median follow-up time (months, IQR)	33.4 (14.9–53.7)
Restenosis ≥70% during follow-up	12 (5.7%)

### Periinterventional inflammatory serum biomarkers

Preinterventional CRP (CRP_pre_) values were not normally distributed and had a median value of 2.1 mg/dl (IQR: 0.0–6.8 mg/dl). According to our local laboratory reference values pathological CRP_pre_ values of >8.0 mg/dl occurred in 37 patients (21.1%). There was a statistically significantly higher median CRP value of 10.4 (IQR: 1.5–23.7 mg/dl) in the group of patients with an ISR in comparison to the group of patients without an ISR (2.0 mg/dl, IQR: 0.0–6.6 mg/dl) during follow up (p = 0.022, see table 2). Postinterventional CRP (CRP_post_) did not differ between the groups (p = 0.314) on univariate level.

**Table 2:** Periprocedural variables.

Variable	No Restenosis	In-Stent Restenosis	p value
	n = 198	n = 12	
Serum parameters			
Leucocyte_pre_ count/µl (mean, SD)	7626 (±2040)	8300 (±3013)	0.283
CRP_pre_ mg/dl (median, IQR)	2 (0–6.6)	10.4 (1.45–23.7)	0.022*
Leucocyte_post_ count/µl (mean, SD)	8526 (±2525)	10433 (±4177)	0.035* †
CRP_post_ mg/dl (median, IQR)	10.1 (4.5–24.9)	9 (3.0–11.2)	0.314
Cholesterol mg/dl (mean, SD)	195 (±49)	192 (±58)	0.885
Triglycerides mg/dl (mean, SD)	148 (±72)	123 (±61	0.439
LDL mg/dl (mean, SD)	133 (±40)	123 (±50)	0.587
HDL mg/dl (mean, SD)	47.8 (±13)	44.8 (±13)	0.620
Interventional parameters			
Predilatation	21 (10.9%)	3 (25.0%)	0.153
Postdilatation	189 (95.5%)	10 (83.3%)	0.125
Multiple stents used	10 (5.1%)	1 (8.3%)	0.485
Stent length (mean, SD)	37.6 (±5.5)	40.7 (±7.0)	0.068* †
Stent width (mean, SD)	7.4 (±1.0)	6.9 (±1.1)	0.019* †
Closed cell stent design	165 (83.3%)	11 (91.7%)	0.695

pre = preprocedural (within 24 hours before CAS), post = postprocedural (within 24 hours after CAS), CRP = C-reactive protein.

*factors included into multiple regression analysis.

† factors remained significant after multiple regression analysis.

Preinterventional leukocyte counts (Leuko_pre_) were normally distributed and reached 7626/µl (±2040/µl) in the group without and 8300/µl (±3013/µl) with ISR (p = 0.283).

The postinterventional leukocyte count (Leuko_post_) showed a significant increase in patients with the occurrence of an ISR during follow-up in the univariate analysis (8526/µl vs. 10433/µl; p = 0.035).

The lipid profile did not differ significantly between both groups.

### CAS-related interventional parameters

In most of the cases (79%) a closed-cell design Carotid WALLSTENT (Boston Scientific, Natick, Massachusetts) was used. The stent cell design did not differ between the groups with and without ISR during follow-up (see Table 2, p = 0.695). There was yet a significantly narrower stent width in the group with the occurrence of an ISR (mean: p = 0.019), as well as a trend towards ISR formation during follow-up in those patients with a longer stent length (p = 0.068). There was no significant association between the use of multiple stents, pre- and postdilation and ISR on the univariate level (Table 2).

### Multivariate analysis

After applying binominal multivariate analysis with variables which were imbalanced on univariate analysis (CRP_pre_, Leuko_post_, stent length and width) and correcting for possible influencing variables such as age, gender and symptomatic status of the carotid artery (symptomatic vs. asymptomatic), the variables Leuko_post_, stent length and stent width remained significant for predicting the occurrence of an ISR (Odds ratio (OR) and 95% confidence interval (CI): OR 1.31, 95% CI 1.02–1.69, p = 0.036; OR 1.25, 95% CI 1.05–1.65, p = 0.022 and OR 0.28, 95% CI 0.09–0.84, p = 0.01, respectively, see table 3).

**Table 3:** Statistically significant results of the multiple regression analysis.

Variable	Odds ratio	95% confidence interval	p-value
Leucocyte_post_/1000/µl	1.31	1.02–1.69	0.036
Stent length [mm]	1.25	1.05–1.65	0.022
Stent width [mm]	0.28	0.09–0.84	0.010

Corrected for: status of the carotid artery stenosis (symptomatic or asymptomatic), age and gender.

## Discussion

The aim of our study was to analyze the effect of periinterventional serum inflammation markers and procedure related technical factors during CAS on the development of an in-stent restenosis during long-term follow-up. Our results suggest that post-procedurally elevated leukocyte count as an inflammatory serum marker is an independent predictor for subsequent ISR. Moreover, the occurrence of ISR is markedly regulated by stent dimensions, i.e. the thinner and longer the deployed stent the more frequently a restenosis could be detected.

The pivotal role of vascular inflammation in the development of ISR has been well documented in the context of coronary artery stenting [11]. ISR is mostly triggered by an endothelial disruption and abrasion which is caused by balloon inflation and stent placement. This vascular injury initiates the release of several mediators leading to adhesion of thrombocytes, neutrophiles and monocytes. These cells, for their part, release vasoactive, thrombogenic, lymphocytic, and mitogen substances, which lead to vasoconstriction, vascular remodelling, neointimal proliferation, thrombosis and inflammation finally resulting in ISR [8], [19]. Assuming a similar pathophysiology of ISR within the carotid arteries, the periinterventional monitoring of such inflammatory markers may be useful for the prediction of ISR. The rate of ISR ≥70% within our study population was 5.7% and occurred early after a median duration of 8.6 months follow-up, suggesting more an intimal hyperplasia than an atherosclerotic burden as the main cause of stenosis.

To identify patients with a higher risk for ISR is of high relevance for clinical routine practice because a tight follow-up in these patients is warranted. The inflammatory biomarker CRP has proven its predictive value of clinical outcome and ISR in patients undergoing coronary and carotid artery stenting [11], [13], [20]. Within our patient cohort, the predictive value of preinterventionally elevated CRP could only be shown on a univariate level whereas it did not remain significant after applying multivariate regression analysis due to interaction effects. Moreover, our data could not support an association between CRP_post_ and the formation of ISR as has been demonstrated previously [21]. This may be attributable to the plasmakinetics of CRP showing a delayed increase after the triggering event [22]. In fact, the most predictive increase of postprocedural CRP for ISR could be detected 48 hours after CAS [21] and this might have been missed since our CRP samples were drawn within 24 hours of the CAS procedure.

In contrast to CRP the leukocyte count is known as an inflammation marker rising immediately and rapidly after injury [23]. This might explain why our results showed a significant association between postprocedural leukocyte count and ISR, but failed to demonstrate a relation between postprocedural CRP and ISR.

Technical and procedural factors during CAS could also play an important role in the development of ISR because injury to the intima of the stented artery may cause a hyperplasia. Usually, the selection of the stent length and width are based on angiographic findings in order to appropriately cover the stenosis. Our data show a significant correlation between stent width and ISR. The wider the stent was the less likely was the occurrence of ISR during follow-up. It is conceivable that a stent with a larger diameter results in a reduced flow-velocity, less turbulences and thus in less frequent ISR. Our data are supported by similar results of Clark et al., demonstrating that an underexpanded stent with a small final lumen had a correspondingly higher risk for ISR during serial intravascular ultrasound follow-up [24].

Moreover, a greater length of the deployed stent yielded a more frequent occurrence of ISR within our patient cohort and might be explained by a consecutive longer lesion length which has repeatedly been identified as an independent predictor for periprocedural complications [25], [26]. Apart from certain anatomical considerations concerning e.g. the distance between stenosis and carotid bulb or the presence of poststenotic kinking, the interventionalist's choice of stent length is mainly based on the length of the stenotic lesion and may represent an overall marker for the carotid plaque burden. Our data support the theory that the risk of vessel injury with subsequent inflammation and possible ISR development seems to be higher in larger and longer carotid plaques because longer stents may have been used and resulted in an extended intimal damage.

Currently, the clinical impact of an ISR is still controversially discussed. The development of an ISR was associated with poor sequelae in a single-center study [27] whereas the SPACE-trial did not find a higher stroke incidence during follow-up in patients with an ISR [28], assuming a benign course of this entity. Even if sufficient long-term data assessing the clinical impact of a re-stenosis after CAS will be available in the future, it is still to be expected that a higher degree of re-interventions even in clinically silent ISR will be performed in these patients which again might be accompanied by a periinterventional risk.

Despite the strengths of our conclusive results we are well aware of some limitations of our study: Notwithstanding the prospective data-collection the current analysis was done in a retrospective manner which could have led to an ascertainment bias. To avoid a statistical bias due to sample size and outcome events the analysis of variables investigated had been restricted. Finally, CAS technique improved noticeably during the past few years which may have biased the results during the recruitment period of the current analysis.

The current data contribute to the growing knowledge of factors, which might influence ISR development and are useful for the future individual patient selection and guidance to achieve an optimal treatment of a carotid artery stenosis. The fact that postprocedurally elevated leukocyte count as well as a longer and thinner stent size are associated with the development of an ISR after CAS highlight the theory that inflammation initiated by vessel injury plays an essential role in ISR formation and encourages a rigorous follow-up of these patients.
